# Engineering MoS_*x*_/Ti/InP Hybrid Photocathode for Improved Solar Hydrogen Production

**DOI:** 10.1038/srep29738

**Published:** 2016-07-19

**Authors:** Qiang Li, Maojun Zheng, Miao Zhong, Liguo Ma, Faze Wang, Li Ma, Wenzhong Shen

**Affiliations:** 1 Key Laboratory of Artificial Structure and Quantum Control, Ministry of Education, Department of Physics and Astronomy, Shanghai Jiao Tong University, Shanghai, 200240, People’s Republic of China; 2 Collaborative Innovation Center of Advanced Microstructures, Nanjing, 210093, People’s Republic of China; 3 Department of Chemical System Engineering, The University of Tokyo, 7-3-1 Hongo, Bunkyo-ku, Tokyo 113-8656, Japan; 4 School of Chemistry and Chemical Technology, Shanghai Jiao Tong University, Shanghai, 200240, People’s Republic of China

## Abstract

Due to its direct band gap of ~1.35 eV, appropriate energy band-edge positions, and low surface-recombination velocity, *p*-type InP has attracted considerable attention as a promising photocathode material for solar hydrogen generation. However, challenges remain with *p*-type InP for achieving high and stable photoelectrochemical (PEC) performances. Here, we demonstrate that surface modifications of InP photocathodes with Ti thin layers and amorphous MoS_*x*_ nanoparticles can remarkably improve their PEC performances. A high photocurrent density with an improved PEC onset potential is obtained. Electrochemical impedance analyses reveal that the largely improved PEC performance of MoS_*x*_/Ti/InP is attributed to the reduced charge-transfer resistance and the increased band bending at the MoS_*x*_/Ti/InP/electrolyte interface. In addition, the MoS_*x*_/Ti/InP photocathodes function stably for PEC water reduction under continuous light illumination over 2 h. Our study demonstrates an effective approach to develop high-PEC-performance InP photocathodes towards stable solar hydrogen production.

The imminent depletion of fossil fuels with the increasing environmental concerns has been stimulating considerable research efforts for clean and renewable energy production over the past decades^1^,^2^,^3^. Hydrogen is one of the most promising high-energy-density green fuels. Photoelectrochemical (PEC) water splitting is a highly attractive means to produce hydrogen from water using abundant solar energy^4^,^5^,^6^. Since the pioneering work of hydrogen generation from water splitting using a titanium dioxide (TiO_2_) electrode^7^, enormous efforts have been paid to find suitable semiconductors for efficient PEC hydrogen generation^8^,^9^,^10^,^11^,^12^. However, the reported solar-to-chemical energy conversion efficiencies are still unsatisfactory. Suitable photoactive semiconductors for efficient solar-driven water splitting must (i) absorb a large portion of light in solar spectrum, (ii) have favorable band edge positions, (iii) possess high stability under PEC operating conditions, (iv) be catalytically active for the hydrogen evolution reaction (HER) or oxygen evolution reaction (OER)^13^, and (v) generate a sufficiently high photovoltage. A single semiconductor material is usually difficult to meet all the above requirements and, therefore, effective combination of materials in functional heterogeneous structures or PEC configurations is necessary^14^,^15^. A common technique is to combine light absorbers with robust water splitting catalysts to enhance photocatalytic activity and stability of the PEC devices.

*P*-type InP is one of the most promising candidates for PEC hydrogen generation because of its direct band gap of 1.35 eV well-matched to the solar spectrum, favorable conduction band position for hydrogen evolution reaction and low surface-recombination velocity^16^. InP photocathodes have been extensively studied^16^,^17^,^18^,^19^,^20^ and recently reported a high water splitting performance (power-saved efficiency of 15.8%)^21^. However, the practical application of InP in PEC schemes is still limited by the two main drawbacks of (i) the poor stability due to self-photocorrosion in electrolyte solution^22^,^23^; and (ii) the inefficient surface catalytic activity for hydrogen evolution reactions. Conformal layers of thin TiO_2_ coatings grown by atomic layer deposition have been wildly employed to stabilize InP^16^,^21^,^24^,^25^, and in conjunction with noble metal co-catalysts like Pt^16^,^21^,^24^ to effect water reduce. Nevertheless, relatively high cost and scarcity of noble metals hindered their large-scale practical applications. Moreover, separated noble metal catalysts on top of InP cannot prevent photocorrosion in InP photocathodes. Although thick layers of noble metals can protect photocathodes, they may also block the underlying InP from effectively harvesting light, and decreases the PEC performances. Therefore, low-cost InP-based photocathodes with efficient and stable PEC performances are desirable.

Mo-based chalcogenides have been recently investigated as both a protection layer and an electrocatalyst for hydrogen evolution reaction due to its excellent stability and high electro-catalytic activity^26^,^27^,^28^,^29^,^30^. Thus, Molybdenum sulfide (MoS_*x*_) has a great potential to enhance both stability and activity for InP photocathodes. Indeed, the previous study has shown that the MoS_3_-modificated InP photocathode exhibits a highly stable and large photocurrent density^20^. However, the onset potential was relatively negative in the reported structure likely related to a small photovoltage produced in the InP/electrolyte. The water reduction onset potential of many semiconductors is inherently low, due to the mismatch of the interface energetics between the semiconductor and the electrolyte^21^. To address the intrinsic limitation, several approaches have been used to tune the relative energetics at the semiconductor/electrolyte interface. For instance, an n^+^ emitter layer can be incorporated on the top surface of *p*-type semiconductor to form a buried junction between the semiconductor and the electrolyte. This buried junction is expected to produce a high photovoltage by effectively decoupling the band bending in the semiconductor from the semiconductor/liquid contact^26^. Lewis *et al*. have showed that the HER onset potential could be improved by ~250 mV by using n^+^ p-Si structure device^31^. In addition to homo-junctions, hetero-junctions with appropriate band positions also function efficiently. A recent work has shown that the introduction of a Ga_2_O_3_ buffer layer between the Cu_2_O and TiO_2_ can shift the onset potential towards an extremely positive value of 1.02 V vs. RHE^32^. Therefore, a suitable buffer layer is believed to effectively increase the photovoltage by forming a better energy band alignment with the semiconductor.

Herein, we introduce a Ti thin layer as a suitable buffer layer between InP and the MoS_*x*_ (denoted as MoS_*x*_/Ti/InP) to achieve an improved photovoltage and a stable photocurrent density. The overall material structure is shown in Fig. 1a. The low work function of the Ti layer affords a high Schottky barrier to *p*-type InP valence band (Fig. 1c). The top MoS_*x*_ further acts as an efficient and robust co-catalyst for hydrogen generation. Compared with pristine InP and MoS_*x*_/InP, MoS_*x*_/Ti/InP exhibits remarkably enhanced PEC performances in terms of onset potentials and photocurrent densities at 0 V vs. RHE. Our study is a first demonstration of MoS_*x*_/Ti/InP for efficient and stable PEC water reduction. Such enhancement can be attributed to an improved surface band bending and reduced charge-transfer resistance at the MoS_*x*_/Ti/InP/electrolyte interface, proved by Mott-Schottky and electrochemical impedance spectroscopy (EIS) analyses.

## Results and Discussion

Figure 2 shows the photoelectrodepositon of amorphous MoS_*x*_ (a-MoS_*x*_) catalysts on a Ti-coated InP electrode (Ti/InP). The cyclic voltammetry (CV) cycles show clear reduction peaks similar to the previous report from Hu *et al*.^33^. Once the CVs were completed, a uniform catalyst layer on top of the electrode can be clearly seen. The CV cycles for photoelectrodeposition and the precursor concentrations in solution are considered to be important for determining the thickness of a-MoS_*x*_ on InP. It is reported that the amount of a-MoS_*x*_ on CNTs increases with the increase of deposition cycles or precursor concentrations^34^. The connection between a-MoS_*x*_ particles and InP is strong enough against ultra-sonification, which is essential for long-term use for PEC hydrogen production.

The morphology of the deposited a-MoS_*x*_ films was examined by scanning electron microscopy (SEM). Figure 3 shows the top view and cross-sectional view SEM images of a ~32 nm thick a-MoS_*x*_ film obtained after 30 deposition cycles. The a-MoS_*x*_ film (Fig. 3a) consists of nanoparticles assembled in a porous morphology with a high specific surface area, which improves its catalytic activities. Furthermore, the a-MoS_*x*_ film uniformly covers the InP surface over a large scale (Fig. 3b). This is also important for protecting InP from oxidation and stabilize it for long time operation.

X-ray photoelectron spectroscopy (XPS) was used to characterize the elemental compositions and their binding energies. The survey scan in Fig. 4a shows that Ti, Mo, and S elements are coexisted in the examined film. The chemical states of Mo and S species in the sample are determined from the high-resolution XPS S 2p and Mo 3d spectra before PEC experiments (Fig. 4b,c). The Mo 3d XPS spectrum features two peaks: Mo 3d_5/2_ at a binding energy of 229.2 eV and Mo 3d_3/2_ at 232.5 eV. These energies are consistent with a +4 oxidation state for Mo as reported previously for MoS_3_^35^,^36^. The peak at lower energy position of 226 eV can be ascribed to the S 2s. XPS S 2p spectrum in Fig. 4c exhibits a complex peak, which can be fitted into three featured peaks. The S 2p_3/2_ at 161.9 eV is attributed to terminal S^2−^, and the S 2p_3/2_ at 163.1 eV can be assigned to bridging S_2_^2−^ and/or apical S^2−^ ligands^37^,^38^,^39^. The peak at higher energy (164.5 eV) may be due to residual sulfur from the electrodeposition reactant^37^. Furthermore, quantification analysis by XPS gives a Mo/S ratio of 1:3.4, which is larger than commonly reported for MoS_*x*_ (*x* ≈ 3). Taken together, the XPS data verifies formation of a-MoS_*x*_ phases. It has been reported previously that the abundant exposure of S_2_^2−^ in amorphous MoS_*x*_ can effectively absorb H with a small free energy, which is advantageous for enhancing the HER activity^40^,^41^. Therefore, it is believed that InP with abundant active S edge sites should give potentially outstanding HER performances. After the PEC stability experiments, the Mo peaks are shifted toward lower binding energies (Fig. 4d). This shift can be related to a partial reduction of the Mo atoms or to a change in their environment. The intensity of S peak around 161.9 eV is also increased compared to that of the peak at 163.1 eV (Fig. 4e). These results agree with previous reports that amorphous MoS_2_ might be the most effective water reduction catalyst^33^.

**Figure 1:**
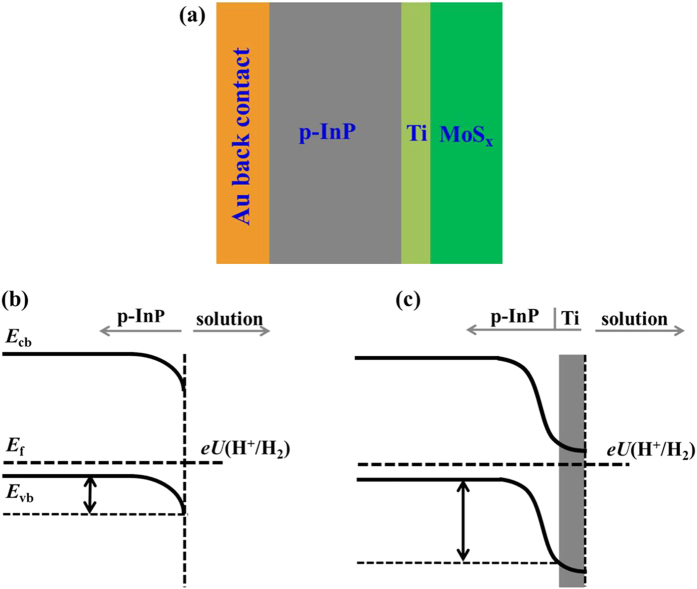
(**a**) Structure of the MoS_*x*_ and Ti-coated *p*-type InP photocathode. (**b,c**) Proposed approximate energy band diagrams of pristine *p*-InP (**b**) and Ti/*p*-InP (**c**) photocathodes in equilibrium with the H_2_/H^+^ redox couple in water in dark. An enlarged band bending is obtained with the Ti-coated *p*-InP in electrolyte due to the low work function of Ti metal.

**Figure 2:**
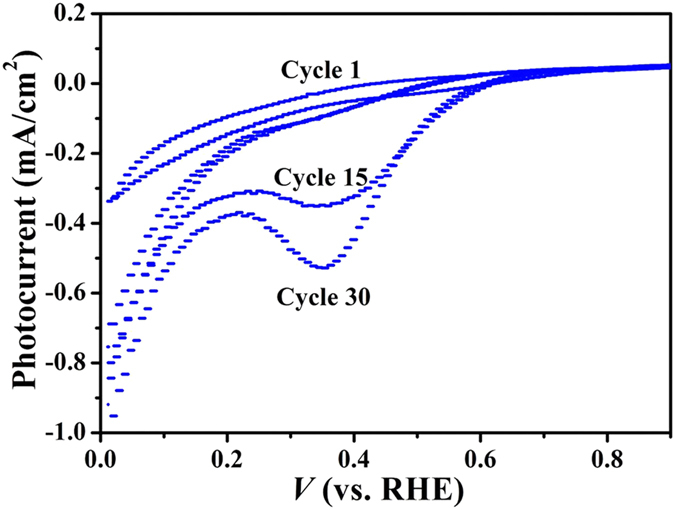
Cyclic voltammetry scans for photoelectrodeposition of MoS_*x*_ on Ti-coated *p*-InP electrodes.

To study the PEC water splitting performance, the photocurrent density vs. potential (*J–V*) curves of the pristine InP, MoS_*x*_/InP and MoS_*x*_/Ti/InP photocathodes were measured in dark and under simulated sunlight illumination (100 mW cm^−2^). All experiments were performed under the same conditions, using 1 M HClO_4_ as electrolyte. The current densities were normalized with respect to the geometrical surface area and reported based on RHE scale. Figure 5a depicts the obtained best-performance *J–V* curves of the three InP-based photocathodes with optimized MoS_*x*_ thickness. In dark, the current density is negligible. Under light illumination, the MoS_*x*_/Ti/InP photocathode exhibits remarkable activity for PEC water reduction. The open-circuit voltage (*V*_*oc*_) and the photocurrent at 0 V vs. RHE (*J*_sc_) of the MoS_*x*_/Ti/InP photocathode under illumination are 0.62 V vs RHE and 15.8 mA cm^−2^, respectively, which both outperforms the pristine *p*-type InP, Ti/InP, and MoS_*x*_/InP. To the best of our knowledge, the PEC performance of our MoS_*x*_/Ti/InP is one of the best reported planar InP photocathodes decorated with noble-metal-free HER catalysts (Table S1). The largely enhanced PEC performance of the MoS_*x*_/Ti/InP photocathode can be attributed to (i) an excellent electro-catalytic performance of MoS_*x*_ which facilitates electron transfer to electrolyte for efficient proton reduction, and (ii) an enlarged band bending formed between Ti and p-InP due to the low work function of Ti (Fig. 1c). The resulting larger band bending favors charge separation and charge transport. Therefore, MoS_*x*_/Ti/InP photocathode afforded a much more positive onset potential than the photocathode without the Ti layer. As shown in Fig. 5a, the PEC difference of InP with and without a Ti layer is clear. The increase of onset potential with Ti modified InP is in consistent with the positive shift of the flat-band potential of the same photocathode (Fig. 6). Moreover, it has been suggested that the Ti overlayer can protect InP from oxidation during the deposition process^26^, which could be another contribution to the PEC improvement of Ti-coated InP photocathodes.

As discussed, a Ti layer on InP can effectively improve the performances, however, it reflects light to reduce light absorption in InP. As a result, the saturated photocurrent density with the MoS_*x*_/Ti/InP photocathodes is smaller than that of the MoS_*x*_/InP without a Ti layer at relatively negative potentials (Fig. 5a). The achieved *J*_sc_ for MoS_*x*_/Ti/ InP is still lower than that of the theoretically photocurrent density of 25–30 mA cm^−2^ for the InP photocathode^42^. Further improvement of the PEC performance is possible by improving light absorption using nanostructured InP photocathodes.

**Figure 3:**
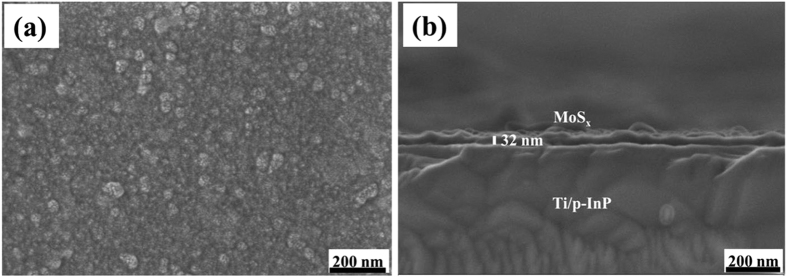
(**a**) A top-view SEM image of a MoS_*x*_/Ti/InP electrode. (**b**) A cross-sectional SEM image of a MoS_*x*_/Ti/InP electrode.

**Figure 4:**
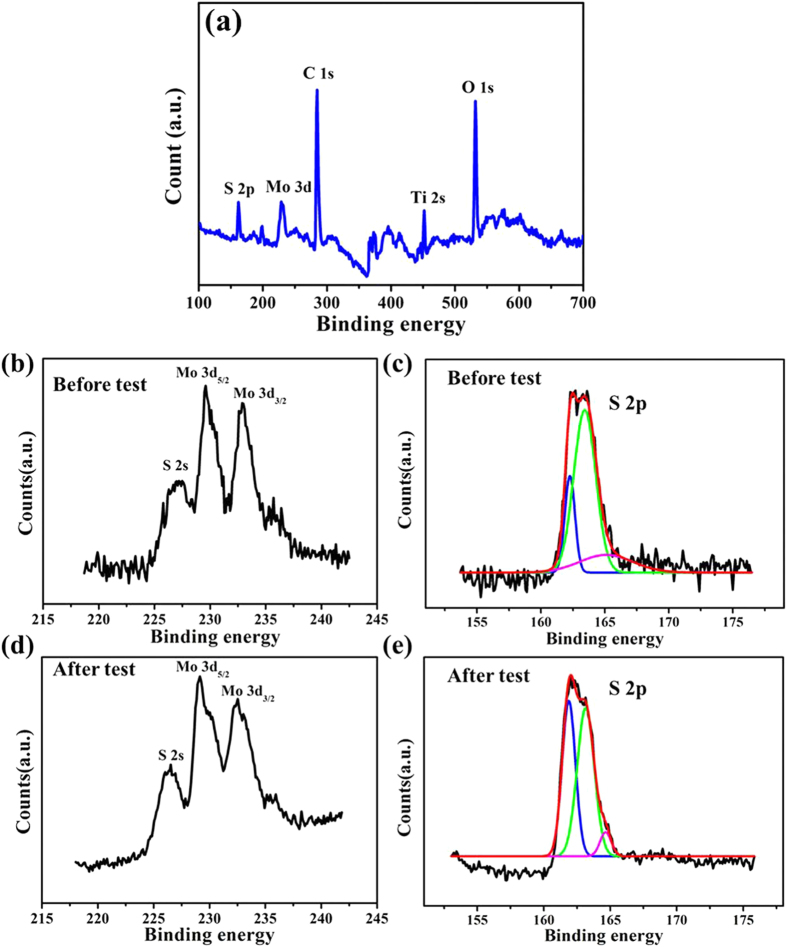
Chemical composition analyses. (**a**) XPS survey spectra. (**b,c**) XPS narrow spectra of Mo 3d and S 2p of as-synthesized MoS_*x*_/Ti/InP electrode. (**d,e**) XPS narrow spectra of Mo 3d and S 2p of the MoS_*x*_/Ti/InP electrode after 2 h PEC test at 0 V vs. RHE in a PEC scheme.

**Figure 5:**
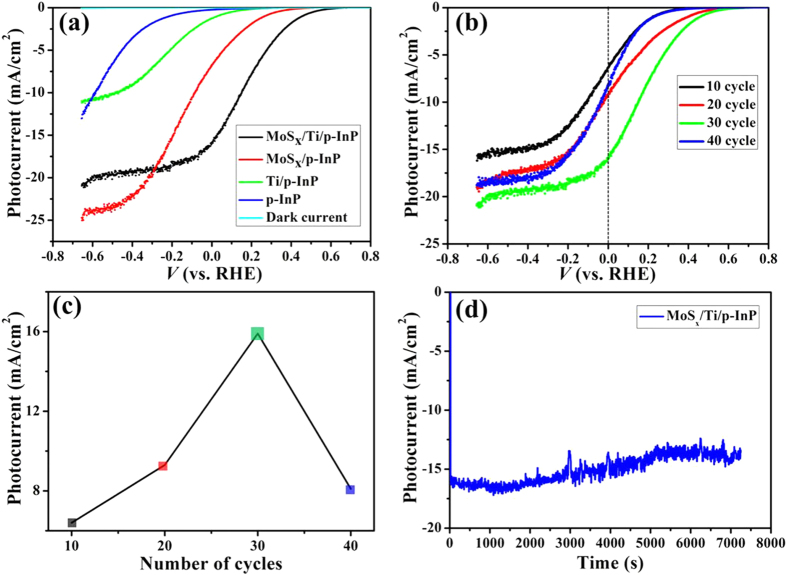
(**a**) Linear sweep voltammetry for pristine InP, MoS_*x*_/InP and MoS_*x*_/Ti/InP photocathodes. (**b**) PEC Performances of the MoS_*x*_/Ti/InP photocathodes with different amount of MoS_*x*_ catalyst. (**c**) Comparison of *j*_*sc*_ at 0 V vs. RHE with different amount of MoS_*x*_. (**d**) PEC stability measurements at 0 V vs. RHE with the best MoS_*x*_/Ti/InP photocathode.

The PEC performance of MoS_*x*_/Ti/InP is determined by the amount of coated MoS_*x*_, which is controlled by the number of CV cycles in the photoelectrodeposition experiments. A suitable amount of MoS_*x*_ on the top surface is usually required to realize optimized PEC performances. We found that 20 and 30 cycles samples have better onset potential than 10 and 40 cycles samples as shown in Fig. 5b. The photocurrent density of MoS_*x*_/Ti/InP loaded with different amount of MoS_*x*_ measured at a potential of 0 V vs RHE is shown in Fig. 5c. MoS_*x*_/Ti/InP with 30 CV cycles for MoS_*x*_ deposition (corresponding to a ~32 nm a-MoS_*x*_ layer with top-view and cross-section images shown in Fig. 3a,b), exhibit the best PEC performance. This is because there is a trade-off between the electro-catalytic activity and light flux. In other words, the electro-catalytic activity will increase with the amount of MoS_*x*_^43^; whereas the light flux absorbed by MoS_*x*_/Ti/InP will be decreased with the increase of MoS_*x*_ amount. Therefore, there would be an optimal amount of MoS_x_ for the best PEC performance.

The PEC stability of MoS_*x*_/Ti/InP photocathodes was investigated at 0 V vs. RHE under continuous simulated solar light illumination (Fig. 5d). PEC photocurrent density remained almost stable over 2 h with only a small decrease from the initial value of 15.8 to ~14 mA cm^−2^ after a 2 h PEC test. The decay of photocurrent density is likely attributed to the degradation of the Ti layer^44^ and/or the H_2_ bubble generation during the stability test. Which is coincides with the visual observation of an excess amount of H_2_ bubbles accumulating on the electrode surface; these bubbles block some active area and interfere with the diffusion of the electrolyte to the photocathode. Moreover, the fluctuation in the photocurrent density is closely related to the bubbling of hydrogen.

To investigate the electronic properties of the pristine and modified InP photocathodes in electrolyte, Mott-Schottky analyses were performed in dark. The flat band potential, *V*_*fb*_, is determined from the interception of the plot of *C*^*−2*^ versus potential curve. The carrier concentration is calculated using the slope of the linear region according to the following equation:


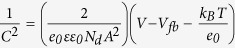


where *C* is the space charge capacitance, *e*_*0*_ is the electron charge, *ε* and *ε*_*o*_ are the dielectric constant of the measured semiconductor and permittivity of the vacuum respectively, *N*_*d*_ is the carrier density, *A* is the measured electrode area, *V* is the applied potential, *V*_*fb*_ is the flat band potential of the measured semiconductor in electrolyte, *k*_*B*_ is the Boltzmann constant, and *T* is the absolute temperature.

**Figure 6:**
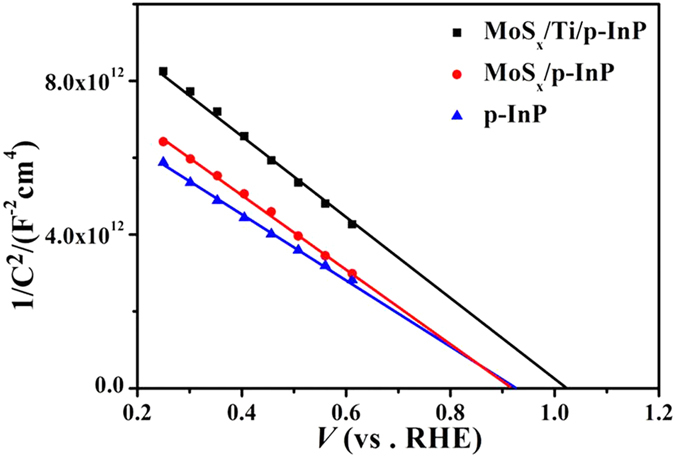
Mott-Schottky plots of pristine InP, MoS_*x*_/InP and MoS_*x*_/Ti/InP photocathodes.

Figure 6 shows that all InP samples have negative slopes in the Mott-Schottky plots, indicating that the samples are all *p*-type semiconductors. The carrier density of InP determined from the slope of the Mott-Schottky plot is ~1.1 × 10^18^ cm^−3^, which agrees with the value from the InP wafer vendor. The *V*_*fb*_ of the pristine InP electrode is estimated to be ~0.92 V vs. RHE close to the previous report^21^. The *V*_*fb*_ for MoS_*x*_/InP is almost the same as that of pristine InP, which is also in consistent with other reports^20^,^27^. However, the *V*_*fb*_ of MoS_*x*_/Ti/InP electrode cathodically shifts from ~0.92 to ~1.02 V vs. RHE, indicating an increased surface band bending, because the low work function of Ti affords a high Schottky barrier to the *p*-type InP valence band^44^,^45^. The increased band bending (Fig. 1b) is advantageous to efficiently separate charges and facilitate charge transfer with a reduced recombination loss. Therefore, MoS_*x*_/Ti/InP provides a much more positive onset potential for hydrogen evolution than the MoS_*x*_/InP electrode as observed in Fig. 5a. This increase is comparable to a different work by enhancing the photovoltage generated by InP in aqueous solutions^21^.

**Figure 7:**
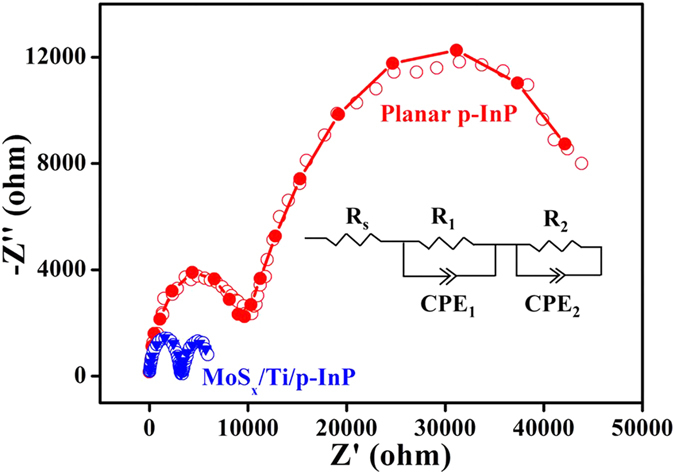
Nyquist plots of pristine InP and MoS_*x*_/Ti/InP photocathodes. The scatter points in the plots represent the original experimental data, whereas the solid lines are the fitted curves using the equivalent circuit model in Fig. 7 (a).

To gain more insight into the principle of the enhancement of PEC performance, electrochemical impedance spectroscopy (EIS) was conducted for different samples under simulated solar light illumination. The circle curves in Fig. 7 are experimental data and solid lines represent fitting results. An equivalent circuit is shown in Fig. 7. As indicated, the equivalent circuit model fitted well with both of the InP electrodes. Two semicircles can be clearly distinguished from Nyquist plots of each sample. The semicircle in the high frequency range is attributed to the depletion layer in semiconductor and the semicircle in the low frequency range is attributed to the capacitance at the semiconductor/electrolyte interface. In our model, R_s_ represents the overall series resistance of the circuit, R_1_ and CPE_1_ correspond to the charge transfer resistance and the depletion layer capacitance in the semiconductor, R_2_ and CPE_2_ are associated with the charge transfer resistance and double layer capacitance at semiconductor/electrolyte interface. Two constant phase elements (CPE) can be visualized as a non-ideal capacitor. It has been suggested that smaller arc radius in Nyquist plot implies a more effective charge separation of electron-hole pairs and a faster interfacial charge transfer^46^. Significantly, MoS_*x*_/Ti/InP show much smaller semicircle, suggesting much smaller charge transfer resistance in MoS_*x*_/Ti/InP, in comparison with the pristine InP electrode.

The fitted data of each element in the model are summarized in Table 1, among which some features deserve attention. The values of R_2_ for MoS_*x*_/Ti/InP and InP are 3013 ohm, 47536 ohm, respectively. The charge transfer resistance of double layer in MoS_*x*_/Ti/InP sample is approximately 16 times smaller than that of pristine InP sample, showing a good correlation with the remarkably enhanced photocurrent density in MoS_*x*_/Ti/InP photocathodes (Fig. 5a). MoS_*x*_ modification reduced the charge transfer resistance and increased the capacitance mainly at MoS_*x*_/Ti/InP/electrolyte interface, i.e. the surface of the photocathode where water reduction reaction takes place. Therefore we can conclude that MoS_*x*_ loading promotes the charge separation and water reduction reaction on the InP surface. This is consistent with the reported results that MoS_*x*_ is an effective electrocatalyst for the hydrogen evolution reaction. In addition, MoS_*x*_/Ti/InP photocathode leads to fast charge transfer in semiconductor bulk, which is attributed to the larger band bending in the photocathode. These results clearly explain the remarkably enhanced performance of the MoS_*x*_/Ti/InP electrode.

In summary, a noble-metal-free MoS_*x*_/Ti/InP photocathode was fabricated by simple deposition processes for solar hydrogen production. MoS_*x*_/Ti/InP exhibited remarkably enhanced PEC performance in comparison with pristine *p*-InP and MoS_*x*_/InP. Specifically, high photocurrent densities and relatively positive onset potentials were observed. The enhancement of PEC performance can be attributed to the excellent electro-catalytic activity of amorphous MoS_*x*_ nanomaterials, which reduces remarkably the charge transfer resistance at the semiconductor/electrolyte interface and increases the kinetics of water reduction. Moreover, the Ti buffer layer was a key component to realize a high photovoltage and to prevent the InP photocorrosion. The performance of MoS_*x*_/Ti/InP was also dependent on the amount of surface MoS_*x*_, for realizing optimal electro-catalytic activity on photocathode surfaces and absorption in InP. More importantly, the photocathodes were stable under continuous simulated solar light illumination over 2 h. Our simple and effective fabrication and surface modification processes are applicable to various photocathodes materials for enhancing their PEC performances.

## Methods

### Preparation of pristine InP samples

The wafers used in this work were one-side polished Zn-doped *p*-type (100)-oriented InP (MTI Corp.) with a carrier concentration of ~10^18^ cm^−3^. To fabricate InP working electrodes, wafers were cleaved into small pieces along the natural (110) cleavage planes. InP pieces were firstly degreased by successively sonicating in trichloroethylene, acetone and methanol for 5 min in each step to remove contaminants followed by washing in deionized water and then drying in N_2_ flow. The pieces were then etched with HF (49%) and H_2_O (1: 10) for 1 min to remove the native oxides from surface. The InP wafers were then thoroughly rinsed using deionized water and dried under a flow of N_2_.

### Sputtering deposition of Ti layers

An ultrathin buffer layer of ~10 nm Ti film was deposited using Denton RF magnetron sputtering. Ti Target (99.95%) was pre-sputtered using RF power (300 W) at a high pressure of 10 mTorr to remove surface impurities prior to the deposition of Ti. The Ti layers were sputtered in an Ar environment. Until the chamber pressure reached 4 × 10^−4^ mTorr, sputtering was conducted. During sputtering, the working pressure inside the chamber was kept at 3.4 mTorr and the deposition power was kept at 200 W. Substrates were held at room temperature in all runs. Deposition rate at this condition was about 2.5 Å/s. InP was held at a room temperature during sputtering. Deposition rate at this condition was about 2.5 Å/s. The sputtered Ti layer protects InP photocathodes from aqueous solutions and transports photogenerated electrons to electrolyte when water reduction takes place.

### Preparation of electrodes

~60 nm thick Au was deposited on the back side of all InP samples using a thermal evaporator. An ohmic contact between Au and InP was formed. Then, high purity Ag pastes were used to attach Au on Cu plates. The copper plates were then covered with epoxy to ensure that only InP pieces were exposed to electrolyte. Copper plates with pieces were pressed in O-rings of electrochemical cells.

### Preparation of MoS_*x*_ on InP electrodes

PEC deposition of MoS_*x*_ catalyst on InP and Ti/InP electrodes was performed under simulated 1 sun illumination. A freshly prepared photocathode was immersed into a 0.5 mM aqueous solution of (NH_4_)_2_MoS_4_ in a 0.1 M phosphate buffer solution at pH 7. The MoS_*x*_ catalyst was deposited by continuous cyclic voltammograms performed using a Princeton potentiostat (PARSTAT 4000) with a saturated Ag/AgCl electrode as a reference electrode and a titanium wire as a counter electrode. The cyclic voltammograms were performed between 0 and +0.9 V vs. RHE at a scan rate of 0.05 Vs^−1^. After this, we carefully washed the InP electrodes by dipping into deionized water several times to eliminate phosphate and [MoS4]^2−^ ions.

### Characterizations and PEC measurements

The sample morphologies were observed using a field-emission scanning electron microscope (FE-SEM, FEI Sirion 200). The surface composition of the sample was analyzed by x-ray photoelectron spectroscopy (XPS, AXIS ULTRA DLD, Kratos, Japan). The binding energy was calibrated with the C1s level of 284.5 eV from surface contaminants. Water splitting performances of photoelectrodes were evaluated in a three-electrode PEC cell with an electrochemical workstation (PARSTAT 4000 model Princeton Applied Research fitted with an impedance analyzer). 1 M HClO_4_ (pH = 0.5) solution was used as the electrolyte. InP, MoS_*x*_/InP and MoS_*x*_/Ti/InP photocathodes, a platinum (Pt) gauze, and a Ag/AgCl electrode were used as the working, counter, and reference electrodes, respectively. All three electrodes were put into a glass cell with a quartz window through which the working electrodes were illuminated from the front sides by a solar simulator (SOLARDGE 700, a 300 W xenon arc lamp equipped with an air mass (AM) 1.5 G filter). The incident light intensity was adjusted to100 mW cm^−2^ by changing the position of lamp relative to that of the electrochemical cell. Before illumination, high-purity N_2_ was purged into cells for 30 min. to remove the dissolved O_2_. The PEC photocurrent density-voltage linear sweeping voltammetry (LSV) curves were measured under interrupted light illumination (100 mW cm^−2^) sweeping form positive potentials to negative potentials with a scan rate of 10 mV s^−1^. The PEC stability test was evaluated under light irradiation at a fixed potential of 0 V vs. RHE. The measured potentials vs. Ag/AgCl were converted to the reversible hydrogen electrode (RHE) according to the equation of *E*_RHE_* = E*_Ag/AgCl_ + 0.059 pH +* E*^0^_Ag/AgCl_, where *E*_RHE_ is the converted potential vs. RHE, *E*_Ag/AgCl_ is the experimental potential measured against the Ag/AgCl reference electrode, and *E*^0^_Ag/AgCl_ is the standard potential of Ag/AgCl at 25 °C (0.1976 V).

The Mott-Schottky and electrochemical impedance spectroscopy (EIS) measurements were performed using the same electrochemical measurement system for PEC measurements. Mott-Schottky measurements were obtained under dark conditions at a frequency of 1000 Hz in a 1 M HClO_4_ solution with a scan rate of 10 mV/s. EIS data were collected under light (100 mW cm^−2^) at +0.1 V vs RHE, with AC perturbation amplitude of 10 mV and a frequency within 10^5^ to 10^−1^ Hz. The EIS spectra were fitted to an appropriate electrical analogue using ZView software.

## Additional Information

**How to cite this article**: Li, Q. *et al*. Engineering MoS*_x_*/Ti/InP Hybrid Photocathode for Improved Solar Hydrogen Production. *Sci. Rep.* **6**, 29738; doi: 10.1038/srep29738 (2016).

## Supplementary Material

Supplementary Information

## Figures and Tables

**Table 1:** Resistances and capacitances determined from Nyquist plots.

Sample	R_S_ (Ω)	R_1_ (Ω)	CPE_1_ (nF)	R_2_ (Ω)	CPE_2_ (μF)
InP	4.37 ± 0.17	10670 ± 310	64.7 ± 5.23	47536 ± 2159	23.5 ± 1.23
MoS_*x*_/Ti/InP	3.92 ± 0.11	3214 ± 20	25.3 ± 1.38	3013 ± 155	178.3 ± 9.31
