# Supplementary material for: Engineering MoSx/Ti/InP Hybrid Photocathode for Improved Solar Hydrogen Production
Source: Sci Rep. 2016 Jul 19;6:29738. doi: 10.1038/srep29738 (PMC4949461; doi:10.1038/srep29738)
Supplement: Supplementary Information [file srep29738-s1.pdf]

Engineering MoS_x/Ti/InP Hybrid Photocathode for Improved Solar Hydrogen Production

Qiang Li[1], Maojun Zheng[1,2], Miao Zhong[3], Liguo Ma[1], Faze Wang[1], Li Ma[4] & Wenzhong Shen[1]

[1]Key Laboratory of Artificial Structure and Quantum Control, Ministry of Education, Department of Physics and Astronomy, Shanghai Jiao Tong University, Shanghai, 200240, People's Republic of China

[2]Collaborative Innovation Center of Advanced Microstructures, Nanjing, 210093, People's Republic of China

[3]Department of Chemical System Engineering, The University of Tokyo, 7-3-1 Hongo, Bunkyo-ku, Tokyo 113-8656, Japan

[4]School of Chemistry and Chemical Technology, Shanghai Jiao Tong University, Shanghai, 200240, People's Republic of China

*Email: mjzheng@sjtu.edu.cn

Table S1 PEC performances of the planar InP based photocathodes for water reduction compared with the reported literatures in recent years.

Ref No.	Substrate	Doping lever	Materials	J_{sc} (mA/cm^2)	V_{oc} (V vs. RHE)
This work	Zn-doped *p*-InP	1~5×10^{18}cm^{-3}	MoS_x/Ti/InP	15.8	0.62
1	unknown	unknown	MoS_2/ InP	6.5	0.4
2	Zn-doped *p*-InP	1.4–1.8×10^{18}cm^{-3}	MoS_3/ InP	14	0.55
3	Zn-doped *p*-InP	5×10^{17}cm^{-3}	Ru/TiO_2/ InP	27	0.5
4	Zn-doped *p*-InP	1.9×10^{17}cm^{-3}	Pt/TiO_2/ InP	29.4	0.63
5	Zn-doped *p*-InP	2.0×10^{17}cm^{-3}	Pt/n^+/ InP	25	0.80

J_{sc} = short-circuit current density; V_{oc} = open circuit voltage

References

1. Kwon, K. C. *et al.* Wafer-scale transferable molybdenum disulfide thin-film catalysts for photoelectrochemical hydrogen production. *Energy Environ. Sci.* (2016).

2. Gao, L. *et al.* Photoelectrochemical hydrogen production on InP nanowire arrays with molybdenum sulfide electrocatalysts. *Nano lett.* **14**, 3715-3719 (2014).

3. Lee, M. H. *et al.* p-Type InP nanopillar photocathodes for efficient solar-driven hydrogen production. *Angew. Chem. Int. Ed.* **51**, 10760-10764 (2012).

4. Hettick, M. *et al.* Nonepitaxial thin-film InP for scalable and efficient photocathodes. *J. Phys. Chem. Lett.* **6**, 2177-2182 (2015).

5. Gao, L. *et al.* High-efficiency InP-based photocathode for hydrogen production by interface energetics design and photon management. *Adv. Funct. Mater.* **26**, 679-686 (2016).
